# Du Strabisme

**Published:** 1842-10-22

**Authors:** M. Clymer


### BIBLIOGRAPHICAL NOTICES.

*Du Strabisme.* Par A. A. Velpeau, Professor de Clinique Chirurgicale à la Faculté de Médecine de Paris, &c. &c. &c. *Supplément aux Nouveaux Elémens de Médecine Opératoire.* A Paris : 1842. 8vo. pp. 180.

*Strabismus.* By A. A. Velpeau, Professor of Clinical Surgery, &. &c. &c. A Supplement to the Elements of Operative Medicine. Paris: 1842.

Until very recently no serious measure had ever been proposed to remedy squinting. The successful application of tenotomy to other deformities, induced Stromeyer, of Dresden, to imagine that it might be successfully applied to the cure of the deformity of squinting, and the operation was accordingly first, successfully, practised by Dieffenbach, on the 29th of October, 1839.

Previous to a description of the various operative methods suggested for the cure of strabismus, Professor Velpeau devotes some pages to the consideration of the anatomy of the appendages of the eye, and more particularly to that of the cellulo-fibrous tissue, which invests more or less completely each of the accessory organs. Before ocular tenotomy came into vogue anatomists had but very imperfectly studied the aponeuroses of the eye; since then they have received every attention, and been described with a minuteness of detail, which leaves nothing to be desired. Having established the anatomical relations of the eye and of its appendages, our author proceeds to describe the various modifications which have been proposed in the operation for squinting. These different methods may be classed under two heads:—A. The ordinary method by dissection, or that of Stromeyer; B. The sub-conjunctival method.

The supposed advantages of the sub-conjunctival method are that it prevents all inflammation and suppuration, by the non-admission of air into the wound. But it is known that the ordinary operation rarely provokes any alarming inflammation, or is followed by any serious suppuration, and moreover, that when they supervene, they need not cause any grave alarm. It is, however, untrue that no air penetrates, by the method of Mr. Guérin, and it is besides very generally followed by infiltration of blood, often sufficiently great to cause an ecchymotic tumefaction of the entire conjunctiva, and even, sometimes, of the whole thickness of the lids. It must, too, be confessed, that all things being equal, the sub-conjunctival method is more difficult for the operator, and more painful, and less sure, for the patient. The only real advantage which Mr. Velpeau conceives can be claimed for it is its not being ordinarily followed by the "ocular polypus," which frequently springs from the bottom of the wound in the Stromeyerian operation from the fifteenth to the thirtieth day. This little vegetation is at most a very trifling inconvenience, and under proper treatment easily disappears, and, if we are not very much mistaken, sometimes succeeds Mr. Guérin's operations.

The modifications of the Stromeyerian operation, numerous as they are, essentially differ so little that our author thinks "that the choice between so many shades, is rather a matter of taste than of necessity." This candid statement is, however, immediately followed by an avowal, the self-complacency of which cannot but excite a smile, at the same time that we may give it our hearty adherence. "4. That, nevertheless, the method, definitively, the easiest and surest, is that one which, for a long while, I have adopted; that is to say, the method in which the blephareirgon keeps the lids apart, whilst the two forceps with serrated points, embrace and raise the muscle at a distance of four to six or eight millimeters, and the conjunctiva, the muscle in its middle portion, with the aponeuroses, are at a single sweep, and at the same time, divided by a pair of good straight scissors; subsequent sections, above and below, dividing all that may offer resistance afterwards."

Two things should occupy the attention of the surgeon after the operation for strabismus;—to prevent accidents, or should they occur, to combat them; and to watch the eye carefully with regard to the maintenance or rëestablishment of a good direction. The simplest means suffice generally for the first of these indications, the subsequent inflammatory action rarely demanding active treatment. The question has arisen whether one of the eyes should not be *covered*, the better to secure repose, and which of the two eyes it should be. Mr. Velpeau thinks that in the majority of cases after the operation, both eyes should be freely exposed to the air, but that generally, the patient should do nothing to fatigue them, keeping his room for three or four days without using his eyes, and afterwards should use them with care, avoiding all irregularities of diet, in fact all imprudences of every kind. In many cases, however, these precautions are useless, and the patients continue their ordinary occupations without inconvenience. In covering the invalid eye, you render it more irritable, without diminishing the liability to inflammation; and you prevent motion, and thus nullify one of the ends proposed. By constant movement the suppleness of the tissues are maintained, and the too immediate union of the divided laminæ to the sclerotica prevented. Where a tendency to deviation in the operated eye persists, then by covering the sound eye, you force it alone to receive the images, and thus to keep itself in the centre of the orbit, by the action of the three intact straight muscles. The operation for strabismus may be followed by incomplete redressment of the eye, by inflammation, and by a fungoid vegetation or polypus, sprouting from the wound. Not only the incomplete division of the muscle, but the existence of the smallest bridle, whether fibrous, cellular, or muscular, will effectually cause the persistance of a vicious direction. But if the tissues be freely incised, and there be subsequent exploration with the blunt hook or the blunt scissors, this will rarely happen. In convergent strabismus, where this deviation has continued, it has been proposed to divide the superior oblique muscle. Mr. Velpeau is opposed to the division of this muscle in such cases, but sometimes divides a portion of the superior and inferior recti muscles, and he thinks, with marked advantage. But it not unfrequently happens that an eye, which, after the operation, remains obstinately convergent, becomes, in the course of four or five days, perfectly straight; and it must be borne in mind that if the sclerotic be too largely denuded, exophthalmia, and separation of the lids may ensue. When, however, this deviation persists, Mr. Velpeau employs compression in the following manner. Small pledgets of lint, or pieces of agaric or soft plaster, placed in the form of a cone at the greater angle of the eye, and confined there by diagonal strips of adhesive plaster, or by the turns of a suitable bandage, prevent the eye from turning towards the root of the nose. The eye itself must not be subjected to compression. Dieffenbach proposed the employment of a thread passed through the conjunctiva, or through the root of the divided muscle, to obviate this anormal deviation, subsequent to the operation for strabismus. Though employed, both by Mr. Velpeau and Mr. Phillips, we cannot conceive its advantages, and its dangers strike us as glaring.

We have already alluded to the vegetation which occasionally appears in the corner of the eye, and to which Mr. Velpeau gives the name of "ocular polypus." We say occasionally appears, because we believe that in at least three-fourths of the cases it will be found wanting. This vegetation, which varies in size from a currant seed to a pea, and has very much the appearance of a strawberry, is formed, our author plausibly imagines, in the following manner : At the same time that the divided edges of the conjunctiva, and the aponeuroses become swollen, the lamellated cellular tissue, which lines the sclerotica, becomes vascularized and softens a little. The whole traumatic surface, which becomes disgorged little by little, is cicatrized from the circumference to the centre. The contact of the lids, which exercise a permanent compression upon a large portion of this wound, leaves a part near the lachrymal caruncle, entirely free. The continual movements of the eye causes the *vascularizable* cellular tissue to be pushed up behind the right portion of the free border of the lids, and it there forms the nidus of the polypus in question. Whatever be its origin, its inconvenience is so slight, as scarcely to demand any treatment, it disappearing generally of itself, in a short time. Its progress, however, may be arrested by the nitrate of silver, or it may be removed, when developed, by the blunt scissors, without pain.

The operation for strabismus is sometimes followed by diplopia, an anormal opening of the eye lids, exophthalmia, and impaired mobility of the eye.

Double vision is common, after the operation, and generally persists, or even sometimes augments, till the second or third week, and then disappears. The separation of the lids, when confined to one eye, is an exceedingly disagreeable deformity, giving to the patient a vacant stare. Mr. Velpeau states that this accident has never supervened on his operations, and he thinks this fact is due to his incising the conjunctival membrane near the cornea. Its cause no doubt is due to too large a dissection of the aponeuroses and tissues of the ball. There is no remedy, and it, therefore, should be avoided. Exophthalmia occurs, not unfrequently, after the operation of strabismus, and produces the most unpleasant effect. Mr. Velpeau, whose operations seem to have been singularly felicitous, has met with it scarcely at all in his own practice. It is irremediable. Mr. Dufresse has recommended compression, exercised on the lids, but we should doubt if with any success. The palpebral suture, recommended by MM. Rognetta and Guérin, is painful to the patient, difficult for the operator, and very doubtful in its result, and should be unequivocally rejected. Sometimes after the operation we notice an incertitude, an inequality in the movements of the eye, and occasionally utter immobility. This last is a sad deformity, giving a distressing, haggard expression to the patient. Unfortunately, as in the other cases, surgery can offer but little chance of cure, and the great object must be to prevent their occurrence. This can only be done by an attentive study of the anatomical arrangement of the contents of the orbit, and more careful study of the manual details of the operation,

An important and delicate task now remains for us to perform—to examine the actual merits of ocular tenotomy, and place its real claims on the profession in a proper light. No operation was ever ushered in under more favorable circumstances: The discovery of an easy, safe and sure method was announced to remedy a deformity which hitherto had completely baffled all the best directed efforts of surgery. Immediate and complete success was fondly imagined and fully promised; all ages and both sexes flocked in crowds to the operators, who were, almost by a miracle, or a fairy spell, to change their destinies. We heard nothing of the dangers, the difficulties and the uncertainty of the operation. Mr. Phillips, the pupil of Dieffenbach, declared that the Surgeon of Berlin had performed upwards of four hundred operations for strabismus, without a single failure or accident; and that he, Mr. P., had operated upwards of one hundred times in St. Petersburg, with invariable success. In France, the floors of the Academies of Sciences and of Medicine were filled with patients who had been operated on the previous evening, and their cures vehemently and rudely proclaimed amidst occasional scenes unworthy of the halls of Science. The political journals of the capital and of the departments were also subsidised, and daily trumpeted forth the successes of certain well known operators. In short, the most disgraceful means were adopted to *allécher les cliens*, and secure a reputation for what promised to be a very lucrative business. Now the fact is, that nearly all the earlier operations in Paris were unsuccessful, and were so, probably, elsewhere in an equal degree. Of Mr. Guérin's four first operations, but one succeeded. All Mr. Velpeau's earlier cases, which we witnessed, failed. Mr. Roux was in the same predicament. Even Mr. Phillips, whose earlier operations *were all successful*, finds the climate of Paris not quite so congenial to the operation as that of St. Petersburg, and out of one hundred cases recently published, he confesses twenty-five failures. Mr. Baugmaster says, that out of fifty-two cases, he cured only thirty-three by the operation. Of seventy-two operations performed at Dresden, forty-five were successful. Mr. Cunier, of Brussels, states, that out of 198 cases, supposed to be cured, in six months after the operation, there were only 70 actual cures. In Belgium, Mr. Dumont declared that the failures were so constant that the operators themselves were discouraged; and Mr. Blairau affirmed at the Medical Society of Ghent that he had never seen a single case of strabismus cured. In 300 cases, Mr. Velpeau declares that only one-half were completely cured. In the other half the cure in one-third was incomplete—that is to say, that there was some slight deviation upwards, or downwards, outwards, or inwards, some immobility, or prominence of the eye, or some discordance in the axes, or in the movements of the two eyes. The remaining two-thirds were complete failures. By failure, Mr. Velpeau means, where there was subsequent permanent deviation in an opposite direction, or a persistance of the original squint, or exophthalmia, &c., or, in a word, where the original deformity remained, or where one even more unpleasant was substituted. With regard to the actual value of the operation for strabismus, Mr. Velpeau abstains from pronouncing any positive judgment; and he thinks that no definitive opinion can be impartially given for some years to come. We do not consider that there is yet sufficient data to justify any rigorous statistical opinion, but we have abundant testimony that the operation in question is not the easy, safe and effectual method it was represented to be, by men, who, though of high professional standing, were, we fear, too much interested by sordid motives. Early enthusiasm has yielded to mature experience and reflection, and repeated failures and lamentable accidents have warned us not too inconsiderately to undertake, or rashly to perform an operation, whose chances of success and failure are nearly equal, and which not unfrequently entails on the patient a worse defect than the one removed.

M. Clymer.

